# Networks of Networks: An Essay on Multi-Level Biological Organization

**DOI:** 10.3389/fgene.2021.706260

**Published:** 2021-06-21

**Authors:** Vladimir N. Uversky, Alessandro Giuliani

**Affiliations:** ^1^Department of Molecular Medicine, Byrd Alzheimer’s Research Institute, Morsani College of Medicine, University of South Florida, Tampa, FL, United States; ^2^Department of Environment and Health, Istituto Superiore di Sanità, Rome, Italy

**Keywords:** network, interaction network, protein-protein interactions, protein structure, protein function, intrinsically disordered proteins

## Abstract

The multi-level organization of nature is self-evident: proteins do interact among them to give rise to an organized metabolism, while in the same time each protein (a single node of such interaction network) is itself a network of interacting amino-acid residues allowing coordinated motion of the macromolecule and systemic effect as allosteric behavior. Similar pictures can be drawn for structure and function of cells, organs, tissues, and ecological systems. The majority of biologists are used to think that causally relevant events originate from the lower level (the molecular one) in the form of perturbations, that “climb up” the hierarchy reaching the ultimate layer of macroscopic behavior (e.g., causing a specific disease). Such causative model, stemming from the usual genotype-phenotype distinction, is not the only one. As a matter of fact, one can observe top-down, bottom-up, as well as middle-out perturbation/control trajectories. The recent complex network studies allow to go further the pure qualitative observation of the existence of both non-linear and non-bottom-up processes and to uncover the deep nature of multi-level organization. Here, taking as paradigm protein structural and interaction networks, we review some of the most relevant results dealing with between networks communication shedding light on the basic principles of complex system control and dynamics and offering a more realistic frame of causation in biology.

## Introduction

The network formalism is probably the most natural way to represent biological systems. Even if in the last decades the analysis of complex networks became a very widespread paradigm to face problems going from macromolecular structures (Di Paola et al., 2013) to genetic regulation circuits (Lopez-Kleine et al., 2013), neuroscience (Petersen and Sporns, 2015), and ecological systems (Bascompte, 2010), this is not a new idea. In 1948 Warren Weaver (1948), one of the fathers of mathematical information theory, sketched a very intriguing synthetic tripartite description of science into problems of “organized simplicity,” “disorganized complexity,” and “organized complexity” with biology located in the last class.

The first class (simplicity) refers to the case of very few elements interacting among them with largely invariant relations. Class 1 problems allow for an extreme abstraction (e.g., a planet can be thought as a dimensionless ‘material point”). The possibility to take into consideration only very few basic (and object independent) features, such as mass and distance, is at the basis of the extreme precision and generality of classical mechanics.

Problems of Disorganized Complexity (class 2) allow for an analogous generalization power by means of a very different style of reasoning. Here, the predictive power stems from the abandoning of the goal to reach the elemental scale shifting to a population level statistical knowledge corresponding to gross averages (like pressure, volume, and temperature are) on a transfinite number of atomic elements. Thermodynamics is the brightest example of this style of reasoning. Both the approaches must fulfill very stringent constraints. Class 1 approach asks for few involved elements interacting in a stable way, class 2 style needs a very large number of identical particles with only negligible (or very stable and invariant) interactions among them. Biological systems, only in a very few cases do satisfy these constraints, so we step into Weaver’s third class (Organized Complexity). Organized Complexity arises whenever many (even if not so many as in class 2) non-identical elements interact with each other by means of links endowed with time-varying correlation strength. The interaction of “non-identical elements” with “varying correlation strengths” corresponds to a network of links (correlations) with variable strength, connecting different nodes that in turn are “non-identical” being themselves networks with variable wiring structure.

Weaver (1948) commented that while science was at home (relying on the usual repertoire of laws and boundary conditions deciding for their application) in both Class 1 and Class 2 phenomena, the overwhelming importance of contextual information with respect to lawful invariant behavior, of Class 3 systems, makes the situation much more uncomfortable. After more than 70 years from Weaver’s article, we made some steps ahead in Organized Complexity studies and the present work deals with some of these advancements. The article is organized as follows: in the first part (biodynamic interfaces), we will discuss the basic principles of the interaction between complex systems, with an emphasis on the need of an intermediate layer shared by the two interacting systems with a partially independent nature with respect to the two interactors. In the second part (the middle way), we will introduce the concept of mesoscopic or “middle-out” organization demonstrating why the “network representation” allows for a natural, hypothesis-free formalization of the meso-scale. The third part will be devoted to the transit of information across a network system and the consequent discrimination from noise of the relevant (signal) perturbations able to “climb-up” or “stepping-down” the multilevel organization. In the fourth part, we will put at work the above considerations analyzing protein-protein interaction (PPI) networks in consideration of the wiring structure of participating proteins. The essay will end with some general conclusions and future possible research trends.

## Biodynamic Interfaces

There is no interaction without information exchange, and there is no information exchange without an efficient communication channel. This channel is exactly what we call “interface.” If Mary calls Peter by means of her smartphone, the establishing of a contact strictly depends on the existence of an electromagnetic field endowed with a band of frequencies devoted to cell phone communication. Peter smartphone corresponds to a very specific frequency modulation of the field that is elicited by the digits Mary composes on her phone and sends on the specific band of frequencies. Consequently, Peter’s smartphone rings and the communication begins. We do not enter into the actual content of communication (that only pertains to Mary and Peter), instead we focus on two crucial points of the process:

1. The existence of a medium (the field) that cannot be considered as a discrete entity with a specific location in both space and time but as a “global feature” covering the space and assuming different values in different locations. The interactors (here the Mary and Peter phones) are causally connected in both directions only because they share the same field. From basic physics we know that a point charge embedded into an electromagnetic field both senses (i.e., is influenced by the field) and modifies (i.e., influences) the field. This is exactly what happens in human-environment interaction, in which environment influences physiology (e.g., toxic effects and sensory information…) and is in turn influenced by humans. Both human beings and environment are complex systems and for their interaction they need a shared interface (Arora et al., 2020).
2. The interface (field) oscillates with a specific frequency, this implies it has both a “spatial” and a “temporal” structure, it is a dynamic interface. The frequency of oscillation is not independent from the spatial features of the interface, more in general, any network system (even a field can be imagined as a grid with some focal points, the “cells” in the case of mobile phones) has characteristic oscillation modes originating from its wiring structure. We will go back on this point when dealing with protein structures “resonating” with specific modes that are the carriers of across levels information. The specificity of the interaction (Mary’s phone call elicits a response only in the Peter apparatus) depends on the resonance phenomenon: an oscillator with a characteristic ω frequency only “recognizes” (e.g., by amplifying its potency) an incoming stimulus with the same (or very similar) frequency.

Both these issues are at work in multi-level organization and, more in general, in biological regulation by networks of networks.

## The Middle Way

The majority of biological explanations and models are made of statements like this: “*gene A provokes the phenotype E by the activation of pathway A-B-C-D-E*,” with B, C, and D being relevant biological players, such as proteins or metabolites, whose concentration (expression) is increased (decreased) or structure is changed (e.g., via posttranslational modifications) by the action of the preceding player. This kind of “pathway” (IF:THEN for informatics) models take for granted the existence of a single “explanatory layer” located at the most microscopic level (gene) that, thanks to a sort of domino effect, ends up into a phenotypic consequence.

This view is in sharp contrast with what we know about complex structured systems, where a multi-layer causality is at work. One of the most clear falsifications of the obliged “bottom-up” character of biological causation, comes from a 1945 article (Fankhauser, 1945) by the German (but United States based) embryologist Gerhard Fankhauser. He considered cell size in polyploid triton larvae that have a doubled chromosome number with respect to their diploid counterpart. The polyploid individuals have a doubled cell size with respect to the diploid ones, notwithstanding that, they have exactly the same dimension of organs and ducts (Fankhauser, 1945). This comes from the fact that the polyploid organism uses half the number of cells, though each cell was itself double in size, to build up its organs. This is crucial for life—the optimization of the caliber of a biological structure (the duct) is finely tuned to fit with the flow of biological fluids (a top-down constraint) and cannot be established by either its constituent cells or the genome. While this is an intuitive tenet (after all, we do not decide the size of our house based solely on the size of the bricks!), it was considered as a largely unexpected finding by Albert Einstein (a colleague of Fankhauser at Princeton) that admitted he was expecting the double size cells should give rise to double size ducts and that the Fankhauser observation pointed to still largely unknown principles (Fankhauser, 1972). The brilliant Fankhauser experiment was largely overlooked and obscured by the successes of molecular biology in the years to come, but it is a clear example of a top-down causative model, in which a “high-level” constraint “slaved” the microscopic cellular/genomic level.

It is important to stress that the “bottom-up only” obsession is not shared by all the biological fields of investigation. Ecologists recognized since many years that the most microscopic level of organization is not necessarily the place where “the most relevant facts do happen.” On the contrary, the most fruitful scale of investigation is where “non-trivial determinism is maximal” (Pascual and Levin, 1999). That is to say, the scale more rich in meaningful correlations between features pertinent to micro and macro- scale or, to use an ecological term: the mesoscopic realm (Cheng et al., 2014).

Non-trivial determinism can be defined in terms of prediction error as (Pascual and Levin, 1999):

Predictionr=21-E/2S2

In the above formula, E is the mean prediction error and S the standard deviation. In the case of a simple linear regression, in which a dependent variable Y must be predicted by an independent variable X, the non-trivial determinism is nothing else than the usual squared Pearson correlation between the two X and Y variables. The formula can be extended to any other situation, in which we wish to predict a system feature Y, both X and Y do not need to represent single variables but any suitable set of information at any definition scale.

The “non-trivial” attribute of determinism stands for the need of “explaining the variance” of the system at hand (the statistic *r*^2^ corresponds to the proportion of variance explained by a model) and not its “average” (or most stable/frequent) pattern: the aim is to account for the actual behavior of the system in both space and time and not to describe a “frozen” ideal configuration.

The individuation (i.e., description of the manner in which a thing is identified as distinguished from other things) of “mesoscopic principles” largely independent from the material constitution of the studied system and only dependent on their relational structure was the theme of an important work written by 1998 Nobel prize in Physics Robert B. Laughlin and colleagues appeared in year 2000 entitled “The Middle Way” that aptly recognized in the discovery of universal mesoscopic principles the next frontier of science (Laughlin et al., 2000). As pointed out by Nicosia et al. (2014): “*Networks are the fabric of complex systems.”* This is why different investigation fields from protein science (Di Paola et al., 2013) to neuroscience (Sporns, 2018) make use of network formalization. The basic idea here is that shared organization rules (i.e., similar wiring patterns) give rise to similar phenomenology, independently of the nature of the constituting elements. In other words, complex network invariants promise to be the place, where to look for universal mesoscopic principles, the viewpoint that maximizes “non-trivial determinism” (Pascual and Levin, 1999).

The Dutch electrical engineer Bernard Tellegen (Mikulecky, 2001) developed a sort of conservation principle of both potential and flux across a network analogous to Kirchoff’s laws. The flux does not need to be an electrical current, and the same holds for the potential, a system represented by a set of nodes linked by edges with a given topology has similar emerging properties independently of the physical nature of nodes and edges. As aptly stressed in Mikulecky (2001), the theorem opens the way to a sort of “network thermodynamics,” whose principles are strictly dependent on the wiring architecture, while largely independent of the constitutive laws governing the single elements.

**FIGURE 1:**
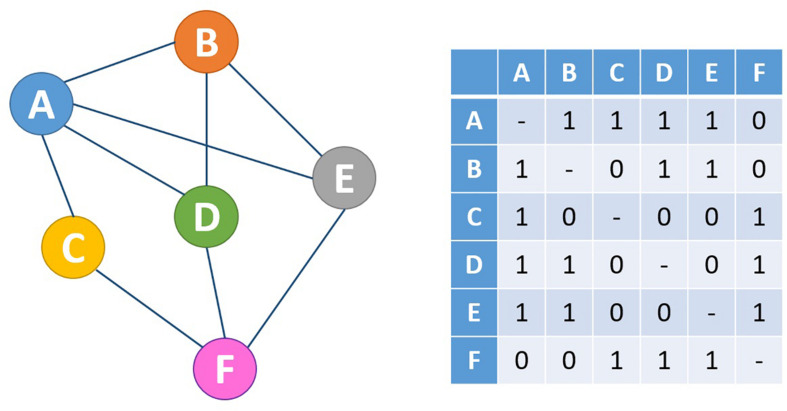
Mathematically, every network **(right)** can be expressed in the form of an adjacency matrix **(left)**. In this case, a network with undirected, unweighted edges is shown, which is represented by a symmetric adjacency matrix containing only the values 0 and 1 to indicate the absence and presence of connections, respectively.

Complex network invariants (Strogatz, 2001) catch the essence of multi-level organization for the simple fact that their estimation merges different level of definition of the system at hand. Mathematically speaking, a network corresponds to a graph, whose entire information is caught by its adjacency matrix (see Figure 1): a binary matrix having as rows and columns the nodes and at each *i*, *j* position a unit value if the *i* and *j* nodes have a direct link between them and 0 otherwise.

**FIGURE 2:**
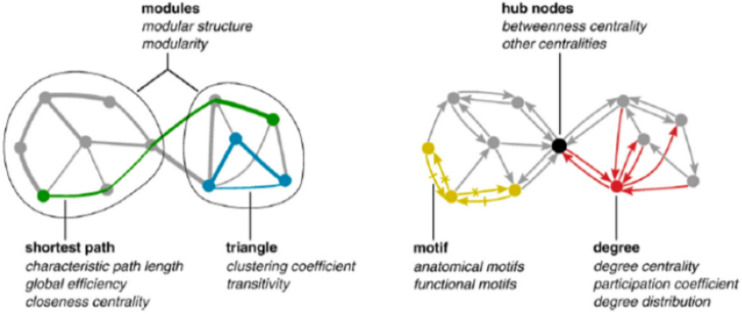
Major features (graph invariants) used to describe networks. It is worth noting the mutual dependence of network descriptors across different scales going from single nodes to the entire network.

Graph invariants are relative to local (single nodes), global (entire network), and mesoscopic (clusters of nodes and optimal paths) levels. The “degree” (how many links are attached to a given node) is a local descriptor, the “average shortest path” (characteristic length) is the average length of minimal paths connecting all the node pairs, and can be considered as a mesoscopic feature, while the general connectivity of the network (density of links) is a global property (Csermely et al., 2013; Giuliani et al., 2014). All these descriptors (and many others) are strictly intermingled across different organization layers. In fact, characteristic length inherits from the “bottom” the information of the single node degree (higher degree nodes have a higher probability to enter into shortest paths), while betweenness (the number of shortest paths passing by a node, thus a strictly speaking a microscopic feature of the network) inherits from the “top” the existence of clusters (modules) of nodes so that a node in between two different clusters A and B is traversed by all the shortest paths linking the A,B node pairs so scoring a high betweenness (Figure 2).

In other terms, describing a system by network formalism implies a multi-level structural representation without the need of “imposing” a particular bottom-up or top-down causative pattern.

## Information Fluxes Across Networks

Biological systems are complex systems that both adapt to their environment and interact with other systems. Provided we are able to find a meaningful formalization in terms of interacting parts, each complex system can be intended as a network. Therefore, it is crucial to understand the peculiarities of information transfer across networks, in order to understand the basic principles of biological organization.

Probably the most straightforward paradigm of information transfer through a network in proteins is the allosteric effect. Allostery is a neologism coming from Greek language, which has to do with the ability of proteins to transmit a signal from one site of molecule to another in response to environmental stimuli. This ability is related to the transmission of information across the protein molecule from a sensor (allosteric) site to the effector (binding or active) site (Hilser et al., 2012). The molecule, hence, perceives ligand binding (or any other micro-environmental perturbation) at distance from the active site, and adapts its configuration accordingly. For example, hemoglobin molecule senses at the allosteric site the partial pressure of oxygen (p[O_2_]): when p[O_2_] is high, the affinity of hemoglobin for oxygen increases and the protein binds oxygen molecules at active site. On the contrary, when p[O_2_] is low, affinity decreases and bound oxygen is released to the cells. This process is crucial for life: in lungs, there is a very high oxygen pressure and the red blood cells containing hemoglobin must catch oxygen molecules that in turn must be released in peripheral tissues (low p[O_2_]) so to make oxidative metabolism possible. How the protein molecule can discriminate such a relevant signal from the continuous motions coming from thermal noise and transmit the information at distance so to reach the active site?

**FIGURE 3:**
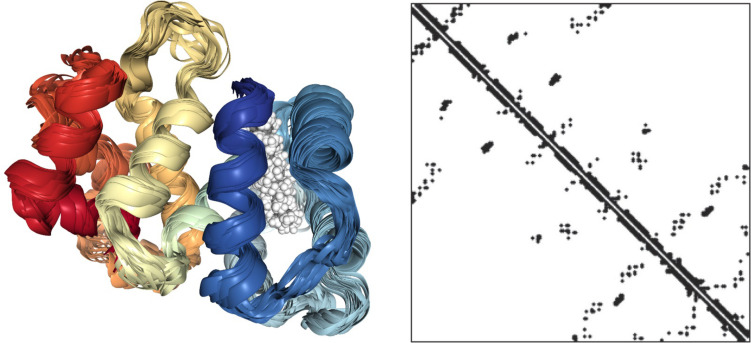
NMR solution structure of myristoylated recoverin in the calcium-free state (PDB ID: 1IKU, left) and correspondent adjacency matrix.

**FIGURE 4:**
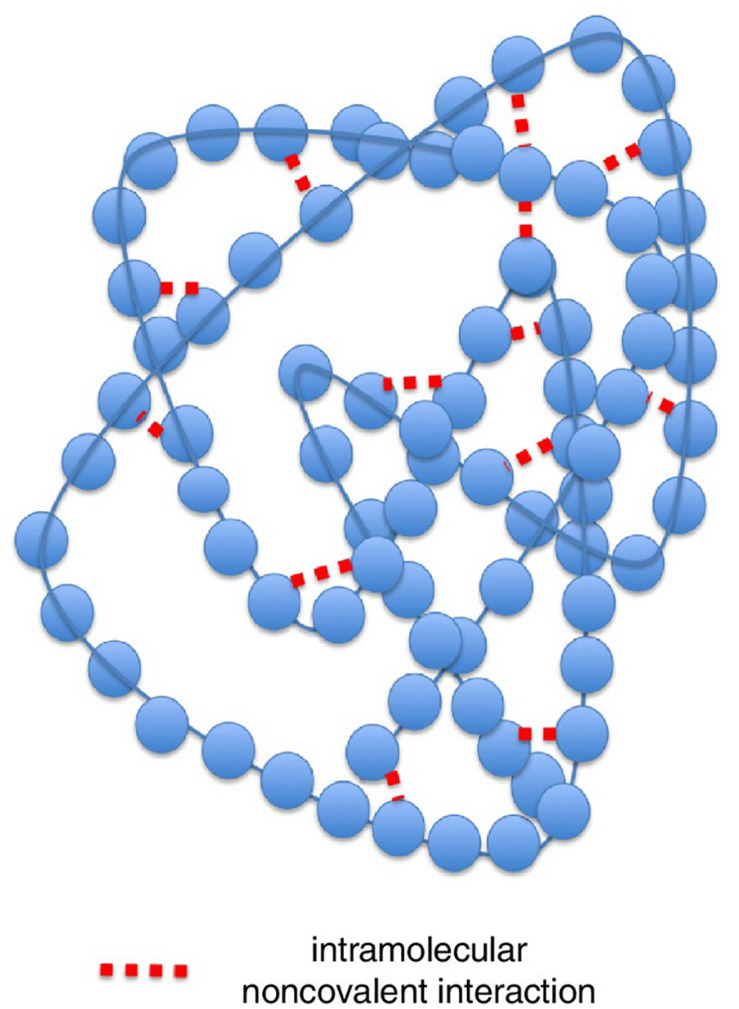
Representation of the intra-protein interactability for a model protein. Here a protein molecule is shown as a bracelet having amino acid residues as pearls and active contacts as dashed red lines. Modified from Di Paola and Giuliani (2015).

To answer this question is useful to consider a protein molecule as a network (Figure 3). In the left panel, the 3D structure of a small protein (recoverin) follows the usual “ribbon” style: the polypeptide chain is represented in terms of contiguous segments of “secondary structure” namely α-helices, irregular structure, and β-sheets (Di Paola et al., 2013). In this particular representation, the parallel segments give an idea of the flexibility of the different tracts of the molecule related to the thermal motion. The right panel represents the same protein in terms of the adjacency matrix of the corresponding network (PCN = Protein Contact Network) whose nodes are the constituent amino acids, while the darkened pixels mark the unit values of adjacency matrix (Figure 1) pointing to an effective pairwise contact between amino acid residues. The amino acid residues are ordered along the protein sequence and the “trivial” contacts between amino acids adjacent along the chain are eliminated. This implies the scored contacts (links of the PCN) correspond to non-covalent intermolecular bonds putting different parts of the molecule into close contact by the action of folding process. This intra-protein interactability is illustrated by Figure 4, where a protein molecule is represented as a bracelet having amino acid residues as pearls and active contacts as dashed red lines.

In PCNs, the shortest paths passing by the network edges mediate concerted motions and energy transmission upon stimulation of allosteric site (Di Paola and Giuliani, 2015; Gadiyaram et al., 2021). The topological metrics of shortest paths (minimum number of links separating two residues) is thus the actual metrics for signaling (Gadiyaram et al., 2021). The discrimination between relevant signals to be transmitted at distance without loss of information and non-informative perturbations to be dissipated without relevant changes in the 3D structure, relies upon two very important mesoscopic network descriptors: “Guimera and Amaral’ *z* and *P* indexes (Guimera and Nunes Amaral, 2005). The index *z* quantifies the number of contacts a given node (amino acid residue in this case) has with other nodes of its own cluster (local contacts), while *P* scales with the number of edges linking the node to amino acid residues pertaining to different clusters.

A perturbation affecting specifically a “high-*P*” node travels a long distance across the network passing by subsequent “high-*P*” nodes and arriving at the destination, thereby supporting allosteric effects. On the contrary, generic (noisy) thermal motion rapidly dissipates distributing across non-directional cycles through intra-module motions.

**FIGURE 5:**
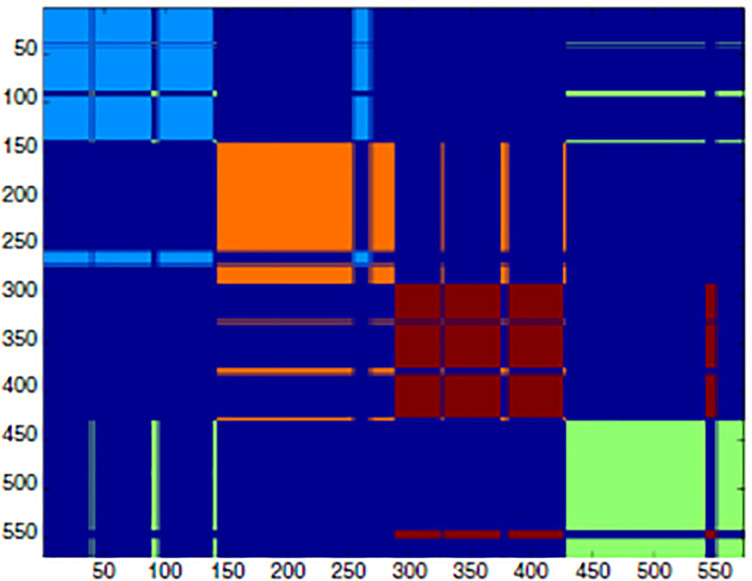
Spectral clustering of hemoglobin. The adjacency matrix is shown as a clustering color map that reports the cluster partition along the sequence. The spectral clustering technique decomposes the space through the adjacency matrix eigenvalues, so that the partition relies on the topological role of residues in the interaction network, rather than on their spatial positioning [modified from Di Paola and Giuliani (2015)].

High-*P* nodes create a “fast lane” for relevant information neatly separated by noise. This is exactly the role of biodynamic interfaces: some proteins (multimeric proteins) are made by distinct chains held together by intermolecular contacts. This is the case of hemoglobin that is made by four distinct polypeptide chains: the allosteric effect ends up into a different re-arrangement of the relative positions of the four chains that go back and forth between two different patterns (R and T for Relaxed and Tense) with high and low affinity for oxygen. The interface between these four chains is made of high-*P* amino acid residues that allow concerted motions among the chains. Figure 5 gives a pictorial description of the situation by showing the adjacency matrix of hemoglobin (Figure 5).

Here, the adjacency matrix of hemoglobin is described by a color code. As usual in such presentation, the axes of the figure report the order of the residues along the chains (each chain contains 150 residues). The dark blue corresponds to the lack of contacts, and the different colors correspond to the four chains. It is evident, the presence of “displaced contacts” in the form of residues that, while pertaining to a given chain (module of the network) have the majority of their contacts with amino acids pertaining to different chains. These “displaced contacts” are the long “whiskers” contacting zones different from their own cluster [e.g., the pale blue line pertaining to the first chain (1-150)] that is in contact with the orange (second chain) module). These whiskers correspond to the high-*P* nodes that generate “something in between” the interacting systems with a “shared ontology” across the interacting systems (polypeptide chains). Very similar models allow for the synchronization of interacting networks thereby passing from single stimulus effects to sustained periodic oscillations.

## Network of Networks: From Single Proteins to Protein-Protein Interactions

Any protein can be considered as a specific network of residue-residue interactions. Importantly, such network consideration works for both monomeric proteins (e.g., aforementioned recoverin and hemoglobin monomer) and oligomeric proteins [e.g., hemoglobin heterotetramer (αβ)_2_]. These and many other similar examples can be used as illustrations of information flow within ordered proteins and ordered protein complexes. In fact, in such cases, protein (protein complex) is characterized by a unique, relatively stable crystal-like 3D structure whose Ramachandran angles vary only slightly around their equilibrium positions with occasional cooperative conformational switches and with almost constant and very specific residue-residue interactions that are relatively fixed in time and space. The stability of such a uniquely folded structure of an ordered protein is defined by the tight packing of its interior achieved by multiple specific residue-residue interactions (Pace et al., 2014). There is very little free space in the protein interior (Richards, 1963; Klapper, 1971; Lee and Richards, 1971), which is closer to a solid than to a liquid (Klapper, 1971), since it is twice as tightly packed as water and possesses a packing density, which exceeds that of closely packed spheres (Pace et al., 2014). This tight packing is achieved during protein folding by burying about 85% of the non-polar side groups, 65% of the polar side chains, and 70% of the peptide groups (Lesser and Rose, 1990), and due to the formation of 1.1 hydrogen bonds per residue (Stickle et al., 1992). This stable structural organization, supported by the numerous crystal structures of proteins solved by X-ray diffraction, resulted in a very common use of terms “unique 3D structure” and “rigid 3D structure” for the description of the structural properties of ordered proteins. Furthermore, the relative rigidity of structures of globular proteins was further supported by their high conformational stability and cooperative folding-unfolding behavior, where, for example, denaturant-induced unfolding was described as a reversible and highly cooperative “all-or-none”-type transition between native and denatured states (Tanford, 1968), and where the temperature-induced melting was shown to be accompanied by the cooperative heat absorption related to the sharp change in the state of a protein on heating (Privalov, 1979, 1982).

However, it is recognized now that considering a protein molecule as a static entity with “rigid 3D structure” and a unchanging PCN is an oversimplification, as proteins are rather dynamic biological systems that have some degree of flexibility, as a matter of fact we observe changes in PCN of apo- and holo-forms and in response to allosteric effectors (Di Paola et al., 2013; Di Paola and Giuliani, 2015). In fact, the importance of conformational flexibility and the need of dynamics for the successful functionality of globular proteins (even enzymes) was emphasized in many studies over the past 65 years or so (e.g., Koshland, 1958; Villa et al., 2000; Agarwal et al., 2002, 2004; Eisenmesser et al., 2002, 2005; Rajagopalan and Benkovic, 2002; Sutcliffe and Scrutton, 2002; Tousignant and Pelletier, 2004; Agarwal, 2005; Yang and Bahar, 2005; Olsson et al., 2006; Frauenfelder et al., 2009). The internal dynamics of enzymes (i.e., movement of their parts including individual amino acid residues, a group of amino acids, or even an entire domain that occurs in a wide range of time-scales, from femto-seconds to seconds) has been suggested to be linked to their mechanism of catalysis (Eisenmesser et al., 2002, 2005; Agarwal, 2005). Furthermore, the existence of conformational sub-states (which were detected based on the atomic displacements involved in the inter-conversion of different local configurations of the same overall protein structure) in globular proteins potentially related to their functional conformational changes and allosteric behavior has been established (Austin et al., 1975; Artymiuk et al., 1979; Frauenfelder et al., 1979; Beece et al., 1980; Frauenfelder and Petsko, 1980; Parak et al., 1981; Hartmann et al., 1982). It was also pointed out that although the entire protein molecule is rather flexible, the flexibility is not homogeneously distributed within a molecule, and some structural parts of ordered proteins are more rigid than others (Ma et al., 1999). Such more rigid parts or structural units (which could be structural domains, sub-domains or any other sub-structure) are typically more compactly packed, have a stronger hydrophobic effect and have a larger stabilizing electrostatic contribution (Ma et al., 1999).

A protein with a set of stable structural units can form a range of conformational isomers, structural peculiarities of which (and corresponding PCNs) would depend on the extent of the overall structural flexibility and the locations of the more flexible joints, whereas, in a protein with unstable structural units, the thermal motions of the backbone could generate an entirely flexible molecule (Ma et al., 1999). Notable, in PCN formalism, the residues devoted to structural stability (high *z*, low *P*) are the less flexible, while the opposite holds for high *P* residues. Obviously, the presence of such structural flexibility changes the PCN perception and transforms its representation from a static mesh into a network with spatio-temporal dynamics, where residue-residue contacts are not fixed in time and space, but change over time. This, in turn, complicates information transmission, which cannot be considered as a passage through a rigid bridge or tunnel anymore, but represents an attempt to cross the river by a suspension bridge in a very windy day.

Furthermore, complications and complexity are not stopped there, as in their functional states, many proteins can be disordered to different degree. In fact, recent years provided solid evidence of the existence of the entirely different class of biologically active proteins, which do not have unique structures as a whole or in some parts. These are intrinsically disordered proteins (IDPs) and hybrid proteins containing ordered and intrinsically disordered protein regions (IDPRs), the existence of which has changed protein science. Such proteins are commonly found in proteomes of all the oganisms in all kingdoms of life and all viral proteomes analyzed so far (Dunker et al., 2000; Ward et al., 2004; Tompa et al., 2006; Krasowski et al., 2008; Shimizu and Toh, 2009; Tokuriki et al., 2009; Pentony and Jones, 2010; Tompa and Kalmar, 2010; Uversky, 2010; Xue et al., 2010, 2012, 2014; Schad et al., 2011; Hegyi and Tompa, 2012; Korneta and Bujnicki, 2012; Midic and Obradovic, 2012; Pancsa and Tompa, 2012; Di Domenico et al., 2013; Kahali and Ghosh, 2013; Peng et al., 2015; Kumar et al., 2021). They have crucial roles in various biological processes and their penetrance increases with the increase in the organism complexity (Dunker et al., 2000; Ward et al., 2004; Oldfield et al., 2005; Uversky, 2010; Xue et al., 2012). As a result, the putative fraction of sequences with predicted long IDPRs (30 residues or longer) increases in the order: Bacteria ∼ Archaea << Eukaryota (Dunker et al., 2000; Ward et al., 2004; Xue et al., 2010; Na et al., 2013; Peng et al., 2015), and this increase in the penetrance of protein disorder is linked to the increased roles of structure-less proteins and protein regions in cellular signaling, regulation, and recognition (Wright and Dyson, 1999; Dunker and Obradovic, 2001; Dunker et al., 2001, 2002a,b; Dyson and Wright, 2002, 2005; Tompa, 2002).

**FIGURE 6:**
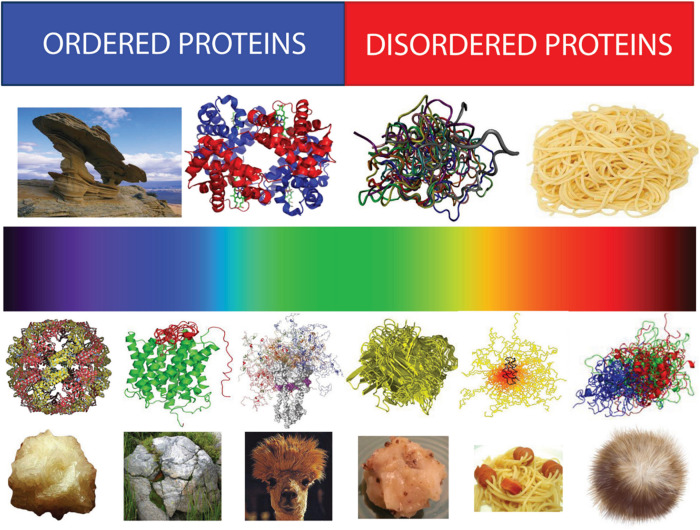
Structural spectroscopy of proteins representing structural heterogeneity of IDPs/IDPRs. Top half: Bi-colored view of functional proteins which are considered to be either ordered (folded, blue) or completely structure-less (disordered, red). Ordered proteins are taken as rigid rocks, whereas IDPs are considered as completely structure-less entities, kind of cooked noodles. Bottom half: A continuous emission spectrum representing the fact that functional proteins can extend from fully ordered to completely structure-less proteins, with everything in between. Intrinsic disorder can have multiple faces, can affect different levels of protein structural organization, and whole proteins, or various protein regions can be disordered to a different degree. Some illustrative examples includes ordered proteins that are completely devoid of disordered regions (rock-like type), ordered proteins with limited number of disordered regions (grass-on-the rock type), ordered proteins with significant amount of disordered regions (llama/camel hair type), molten globule-like collapsed IDPs (greasy ball type), pre-molten globule-like extended IDPs (spaghetti-and-sausage type), and unstructured extended IDPs (hairball type). Adapted from Uversky (2013a).

One of the characteristic features of IDPs/IDPRs is their exceptionally complex and heterogeneous spatio-temporal structural organization, where different parts of a molecule are dynamically ordered (or disordered) to a different degree (Figure 6). In fact, within the highly dynamic conformational ensembles of IDPs/IDPRs one can find foldons (independent foldable units of a protein), inducible foldons (disordered regions that can fold at least in part due to the interaction with binding partners), inducible morphing foldons (disordered regions that can differently fold at interaction with different binding partners), non-foldons (non-foldable protein regions), semi-foldons (regions that are always in a semi-folded form), and unfoldons (ordered regions that have to undergo an order-to-disorder transition to become functional) (Uversky, 2013a,b,c, 2015, 2016a,b, 2019a,b; Jakob et al., 2014; Deforte and Uversky, 2016), whose distribution is constantly changing over time (Uversky, 2013c, 2016c).

This behavior of an IDP/IDPR as a highly frustrated system without single folded state, is reflected in its free energy landscape, which is relatively flat, lacks a deep energy minimum seen in the landscape of an ordered protein, and represents instead a “hilly plateau,” with multiple local minima corresponding to a multitude of conformations and multiple hills that correspond to the forbidden conformations (Uversky et al., 2008; Turoverov et al., 2010; Fisher and Stultz, 2011). Such energy landscape is extremely sensitive to different environmental changes that can modify landscape in a number of very different ways, making some energy minima deeper and some energy barriers higher. This explains the conformational plasticity of an IDP/IDPR, its extreme sensitivity to changes in the environment, its ability to specifically interact with many partners of different nature, and to fold differently as a result of these interactions (Uversky, 2013c).

Obviously, intrinsic disorder plays a crucial role in the organization of the intra-protein networks. In fact, the aforementioned exceptionally complex and heterogeneous spatio-temporal structural organization of a protein molecule with all its foldons, inducible foldons, inducible morphing foldons, non-foldons, semi-foldons, and unfoldons can be presented in the form of an intra-protein network, where residues are involved in transient or more stable conformational interactions. This network is highly dynamic and extremely sensitive to the environment and interaction with partners. Therefore, the aforementioned sensitivity of IDPs to the subtle changes in their environment and capability to fold, often differently, at interaction with binding partners or differently respond to different post-translational modifications (PTMs) or other stimuli, can be considered as a kind of condition-driven rewiring of their intra-molecular networks, where new paths (new connections) can emerge in a condition-specific manner. It is worth noting the strong resemblance of IDPs with the features of biodynamic interfaces we sketched above: this is fully consistent with their role of taking care of physiologically relevant interactions.

**FIGURE 7:**
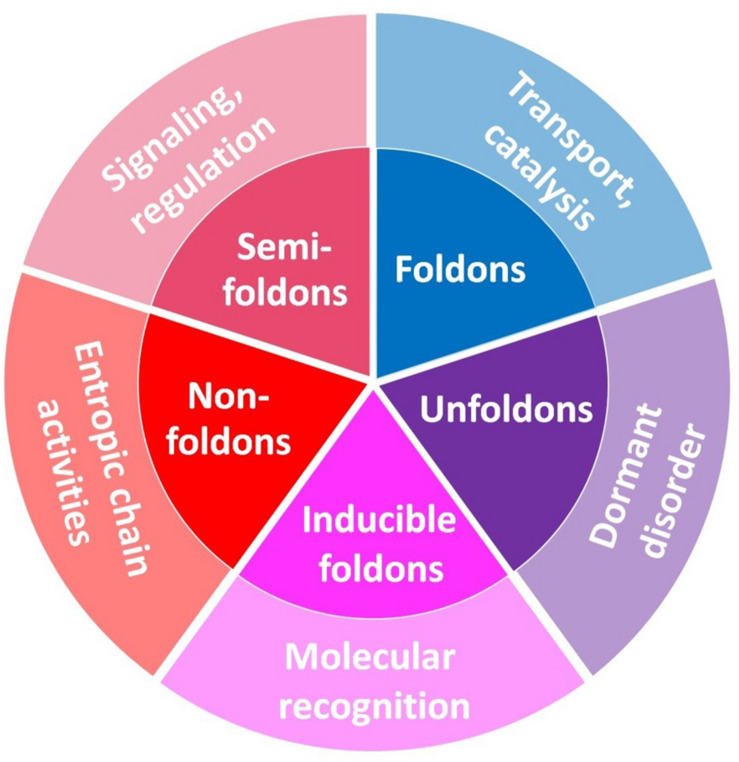
Schematic representation of the mosaic nature of the protein structure–function space. It should be noted that “Dormant disorder” is different from the other “outer-ring” functional grouping because the corresponding segment does not describe a particular functional group but rather represents the means by which the functionality is achieved. Adopted from Uversky (2015).

Therefore, this complex structural organization of IDPs/IDPRs defines their exceptional multi-functionality and serves as a foundation for “protein structure-function continuum” model, where protein exists as a dynamic conformational ensemble comprised of interchanging foldons, inducible foldons, inducible morphing foldons, non-foldons, semi-foldons, and unfoldons and containing multiple proteoforms (conformational/basic, inducible/modified, and functioning) characterized by a broad spectrum of structural features and possessing various functional potentials (Uversky, 2016a, 2019a,b) (see Figure 7).

From the viewpoint of information flow, multi-functionality of such highly dynamic conformational ensembles can be understood if they are depicted as inter-converting ensembles of multi-component systems (networks), whose configurations show extreme sensitivity to the environment. Constituents of these networks are the aforementioned foldons, inducible foldons, inducible morphing foldons, non-foldons, semi-foldons, and unfoldons, which exist transiently (“now you see me, now you don’t”) and define dynamic nature of the network by forming transient contacts with other constituents in the environment-dependent manner. All this places IDPs/IDPRs in the category of the “edge of chaos” systems that operate in the region of maximal complexity (i.e., in a region between order and complete randomness or chaos), where even small changes in the environment might generate large and diversified changes in protein structure and function (Uversky, 2013c, 2019a), defining the ability of a system to differently channel information and to behave as moving staircases in the Hogwarts Castle.

Therefore, a protein molecule represents a complex system that exists as a dynamic, multilevel network of networks. In fact, one can represent a protein molecule as nesting doll (Matryoshka) of the networks of increasing size. Here, at the lowest level, different segments of polypeptide chain form secondary structure elements that represent local networks of hydrogen bonds and residue-residue interactions. The next level of the network is formed by interactions between the elements of secondary structure, which are local networks themselves. This generates foldons, inducible foldons, inducible morphing foldons, non-foldons, semi-foldons, and unfoldons. Next, interactions between these second-tier networks generate higher level networks, proteins domains. Finally, a functional monomeric protein represents seemingly highest level network that includes inter-domain interactions and interactions between domains and second-tier networks. However, formation of an oligomeric protein (and engagement in the temporary protein-protein interactions) would require a new level of inter-subunit interactions, where the inter-protein interaction network might include interactions between the networks of various lower levels.

Despite being a complex system with a complex fate, a single protein is not life *per se*, while protein-protein interactions (PPIs) and their networks are the core of biological regulation. Biological PPI networks belong to the category of the “scale-free” or “small-world” networks, which are neither completely regular (i.e., networks, where each node has exactly the same number of links) or completely random (Erdös and Rényi, 1960). An example of the random networks is given by the highway system, in which despite the random placement of links most nodes have approximately same number of links (Erdös and Rényi, 1960; Barabasi and Bonabeau, 2003). Because the nodes follow a Poisson distribution with a bell shape, such a system almost do not have nodes that have significantly more or fewer links than the average (Barabasi and Bonabeau, 2003). Topology of the PPI networks (as well the airline routes, the author-collaboration network, the metabolic network, gene network, the protein domain network, social networks, and the World Wide Web) is different, as they have hubs, with many connections, and ends, that aren’t connected to anything but a hub (Watts and Strogatz, 1998; Goh et al., 2002). Scale-free networks combine the local clustering of connections characteristic of regular networks with occasional long-range connections between clusters, as can be expected to occur in the random networks. As a result, as a whole, such network has a power-law distribution of the number of links connecting to a node, with some popular nodes possessing a very large number of connections to other nodes, and with the most nodes having just a few (Barabasi and Bonabeau, 2003). Such popular nodes, known as hubs, might have hundreds, thousands or even millions of links depending on the type of network being described. It has been emphasized that from this perspective, the network appears to have no scale (Barabasi and Bonabeau, 2003), and in such scale-free networks, the distance between nodes also follows a power-law distribution (Barabasi and Albert, 1999). This defines the “small world” nature of these networks, as the average distance between two vertices in scale-free network is very small relative to a highly ordered network (e.g., regular lattice), but clustering coefficient is large. As a result, although most nodes are not neighbors of one another, they can be reached from every other node by a small number of steps, since the neighbors of any given node are likely to be neighbors of each other (Watts and Strogatz, 1998) (e.g., in a social network, the small world phenomenon is reflected by a short chain of acquaintances needed to link strangers).

Due to their scale-free nature, PPI networks contains several hubs, which are multitasking proteins that have multiple links. Binding promiscuity of hubs is mostly determined by the intrinsic disorder phenomenon (Dunker et al., 2005; Dosztanyi et al., 2006; Haynes et al., 2006; Oldfield et al., 2008; Hu et al., 2017). In fact, some protein hubs are disordered as a whole, others are hybrid proteins containing both ordered and disordered regions, and very few hubs can be highly structured proteins. Many (but not all) interactions of hybrid hubs are mapped to their IDPRs (Dunker et al., 2005; Uversky and Dunker, 2010), whereas the binding regions of the partners of ordered hubs are intrinsically disordered (Bustos and Iglesias, 2006; Radivojac et al., 2006). These observations clearly indicate that hub proteins commonly use disordered regions (either their own or of their binding partners) to bind to multiple partners (Uversky et al., 2005; Dosztanyi et al., 2006; Ekman et al., 2006; Haynes et al., 2006; Patil and Nakamura, 2006; Singh et al., 2006). The presence of inducible foldons within the conformational ensembles of hubs allow them to (at least partially) fold at interaction with binding partners, whereas the presence of inducible morphing foldons defines the capability of hubs to fold differently at interaction with different partners. All this creates the means for binding promiscuity of hub proteins that relies on intrinsic disorder and related binding-induced disorder-to-order transitions enabling one protein to interact with multiple partners (one-to-many signaling) or to enable multiple partners to bind to one protein (many-to-one signaling) (Dunker et al., 1998).

With respect to the temporal structure of the PPI networks and the roles of intrinsic disorder in maintaining network topology, some proteins have multiple simultaneous interactions (“party hubs”), while others have multiple sequential interactions (“date hubs”) (Han et al., 2004). From a functional perspective, date hubs may connect biological modules to each other (Hartwell et al., 1999), whereas party hubs may form scaffolds that enable the assembly of functional modules (Han et al., 2004). As far as information flow is concerned, PPI network represents a clear example of the “network of the networks of the networks” concept, as it is formed by the interacting Matryoshkas, each being a network of networks itself. Due to the presence of high-*P* and low-*P* nodes and high sensitivity to environment, the topology of a protein PCN (at least PCNs of IDPs/IDPRs) is likely to be described as dynamic inter-converting scale-free networks with the characteristics of the edge of chaos systems, where information can be channeled to different nodes depending on the peculiarities of the protein environment or due to the introduction of post-translational modifications (PTMs). This, in turn, makes PPI network a higher level dynamic non-linear system of the inter-converting scale-free networks possessing the edge of chaos features. This also defines the ability of PPI networks to show the peculiar chaos signature named “butterfly effect,” where a small change in the state of one component of one Matryoshka (e.g., conformational changes induced by binding of a ligand or PTM of a region in one of proteins) can result in large differences in later states (i.e., leading to initiation of different cellular responses). This feature can be considered as the structural counterpart of a “bottom-up” causative chain where a seemingly minor perturbation (e.g., point mutation, ligand binding at a specific receptor, or PTM) gives rise to macroscopic effects. Here it is in action a “permissive” and not an “instructive” (as often implicitly assumed) causative model: the incoming stimulus does not embed the “instructions” for the subsequent process, it only impinges over a “permissive” context (the particular network structure) allowing for the subsequent signal amplification.

## Conclusion

Protein molecules are the most elementary complex systems, lying in the borderline between simple and complex systems physics (Frauenfelder and Wolynes, 1994), they present the basic features of “Weaver organized complexity” (Weaver, 1948): multiple stable states, wiring structure changing in time, adaptation to changing environmental conditions. All these features are acquired by means of biodynamic interfaces (Arora et al., 2020) that, in the case of protein molecules, can be traced down to “high-*P*” residues (and consequently by IDP/IDPR elements). Such features are amplified at the next organization level (PPI), where the same basic principles hold but at an higher level of complexity and, consequently allowing for a much wider repertoire of possible configurations. A coarse grain estimation of the possible “allowed interfaces” between the 25,000 yeast proteins (a very low number with respect to the more than 100,000 protein species of human cells) gives the astronomical number of 10^7200^ (Tompa and Rose, 2011). Notwithstanding that, we observe a relatively low number of “allowed configurations” out of the transfinite number of possible ones: e.g., the actual estimates of “different cell kinds” each with a specific asset of protein-protein interaction pattern tells us of only 411 different human cell types (Vickaryous and Hall, 2006). This dramatic collapse of the number of discrete phenotypes starting from a huge variety of “solutions” at the “bottom of the scale” asks for very strict thermodynamic-like constraints granting for multiple “phenotypically equivalent” solutions at the molecular scale.

The “two-way” interactions between PCN and PPI uncovers some empirical organization principles of the multi-layer networks-of-networks organization of life, here we suggest two of these seminal principles:

1. The “between domains” communication is mainly the duty of “flexible elements.” This creates a partition between structure preserving “conservative” nodes and “creative flexible elements” at each organization layer (Csermely, 2008). This separation is at the basis of biological evolution: it is not by chance that the “structure preserving” amino acid residues are the most conserved, while allosteric signaling are much more prone to mutations along evolutionary scale. Leander et al. (2020) by use of deep mutational scanning, elucidated the molecular basis and underlying functional landscape of allostery. The authors showed that allosteric signaling exhibits a high degree of functional plasticity and redundancy through myriad of mutational pathways. Residues critical for allosteric signaling are poorly conserved, while those required for structural integrity are highly conserved. This result seems at first sight paradoxical: evolution seems to preserve fold over function. But this conundrum is only apparent, if we think that allostery (and, more in general, communication among different layers/domain) has a distributed nature. The presence of multiple equivalent solutions to the thermodynamic conditions of cooperativity (i.e., the collapse to very few phenotypic forms at higher levels) guarantees a much higher resilience (multiple equivalent solutions) with respect to a fine-tuned much more deterministic solution. In the same way, the multiplicity of “quasi-equivalent” communication channels allows for a much more rapid adaptation to a continuously changing environment.
2. Any system made by interacting parts and constrained in a finite size environment oscillates. The frequency of such oscillations roughly (inversely) scales with the size of the system at hand. The entrainment of two oscillators with similar frequencies is the basis of resonance phenomenon provoking a huge amplification of the combined output signal. Resonance phenomena are present at every level of biological organization (Lerner et al., 2018; van der Groen et al., 2018) and are at the basis of the new (and very promising) research avenue of “allosteric drugs” that are able to “generalize” an incoming pharmacological stimulus thanks to resonance phenomena similar to what happens in musical instruments (Zhang et al., 2018; Ni et al., 2019). In a recent work, we found that an ensemble of interacting proteins made of many IDP/IDPR elements was able to greatly enhance the global phenotypic plasticity of yeast cells (Camponeschi et al., 2021). This is an example of a microscopic level stimulus made evident at the macroscopic phenotypic scale thanks to resonance phenomena with external oscillatory stimuli.

All in all, we can affirm the exploration of networks-of-networks can promote a new integrative view of biology at both theoretical and applicative levels. In our opinion, the “bottom-line” of such hierarchy constituted by the analysis of protein and protein complexes, is a perfect playground for generating organization principles universally valid for different organization scales. The “Middle Way” (Laughlin et al., 2000) attitude shifts the “shared foundation of different sciences” from the recognition that “*all the entities are made of the same fundamental particles*” (orienting the various “theories of everything” flourished in the last century) to the statement “*all the entities can be considered as networks of interacting parts*” (Giuliani, 2021). This shift implies the “Universality” of mesoscopic organization principles and the consequent presence of the same wiring rules and emerging properties at different organization layers. This is why, protein science, with its unique mixture of plenty of good quality data and the natural link to a baseline of established chemico-physical properties coming from the adjacent “organized simplicity realm” (Frauenfelder and Wolynes, 1994), is a privileged vantage point for initiating a new avenue of biological research.

## Author Contributions

VU and AG: conceptualization, literature search and analysis, writing – original draft preparation, and writing – reviewing and editing. Both authors contributed to the article and approved the submitted version.

## Conflict of Interest

The authors declare that the research was conducted in the absence of any commercial or financial relationships that could be construed as a potential conflict of interest. The reviewer GH declared a past co-authorship with one of the authors AG to the handling editor.
